# Key players in regulatory RNA realm of bacteria

**DOI:** 10.1016/j.bbrep.2022.101276

**Published:** 2022-05-10

**Authors:** Gowthami Mahendran, Oshadhi T. Jayasinghe, Dhanushika Thavakumaran, Gayan Mirihana Arachchilage, Gayathri N. Silva

**Affiliations:** a Department of Chemistry, University of Colombo, Colombo, Sri Lanka; b Howard Hughes Medical Institute, Yale University, New Haven, CT, 06520-8103, USA; c Department of Chemistry and Biochemistry, University of Notre Dame, IN, 46556, USA; d Department of Biochemistry and Molecular Biology, Center for RNA Molecular Biology, Pennsylvania State University, University Park, PA, 16802, USA; e PTC Therapeutics Inc, South Plainfield, NJ, 07080, USA

**Keywords:** sRNA, Riboswitch, ncRNA, RNA thermometer, Regulatory RNAs

## Abstract

Precise regulation of gene expression is crucial for living cells to adapt for survival in diverse environmental conditions. Among the common cellular regulatory mechanisms, RNA-based regulators play a key role in all domains of life. Discovery of regulatory RNAs have made a paradigm shift in molecular biology as many regulatory functions of RNA have been identified beyond its canonical roles as messenger, ribosomal and transfer RNA. In the complex regulatory RNA network, riboswitches, small RNAs, and RNA thermometers can be identified as some of the key players. Herein, we review the discovery, mechanism, and potential therapeutic use of these classes of regulatory RNAs mainly found in bacteria. Being highly adaptive organisms that inhabit a broad range of ecological niches, bacteria have adopted tight and rapid-responding gene regulation mechanisms. This review aims to highlight how bacteria utilize versatile RNA structures and sequences to build a sophisticated gene regulation network.

## Highlights

• The three major classes of prokaryotic ncRNAs and their characterized mechanisms of operation in gene regulation.
• sRNAs emerging as major players in global gene regulatory networks.
• Riboswitch mediated gene control mechanisms through on/off switches in response to ligand binding.
• RNA thermo sensors for temperature-dependent gene expression.
• Therapeutic importance of ncRNAs and computational approaches involved in the discovery of ncRNAs.

## Graphical abstract

**Figure:**
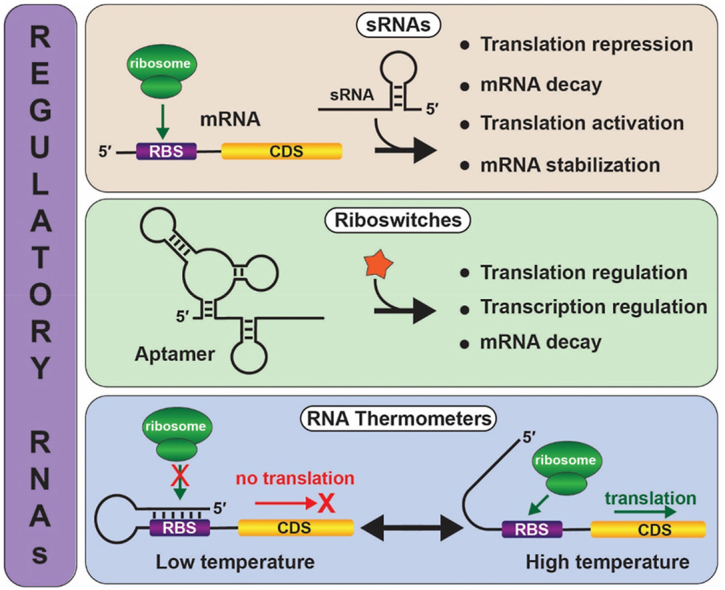

## 1 Introduction

The discovery and characterization of regulatory roles of RNA over the years have established that the role of RNA is not only limited to the central dogma. The functional roles associated with gene regulation that were conventionally reserved for proteins are now understood to be played by RNAs as well. Regulatory RNAs have been found to operate at all levels of gene regulation ranging from transcription initiation to protein stability and activity, in prokaryotic as well as in eukaryotic cells [[1], [2], [3], [4], [5]]. A wide range of physiological responses is found to be modulated through regulatory RNAs. For example, regulatory RNAs are found to play vital regulatory roles cognate to many fundamental cellular processes, such as metabolism and stress responses which have made them eligible for use as attractive therapeutic targets and tools [6,7]. This review aims to highlight how and where regulatory RNAs participate in gene regulation with specific emphasis on small RNAs (sRNAs), Riboswitches and RNA thermometers characterized in bacteria.

RNAs that are possible players of fine-tuning the expression of other genes were discovered in the 1980s. The most well characterized and functionally diverse regulatory RNAs are known as non-coding RNAs (ncRNAs) which are transcribed independently and do not code for proteins [[8], [9], [10], [11], [12]]. Most of the ncRNAs of prokaryotes are transcribed from intergenic regions and antisense strands [13]. However, some ncRNAs are transcribed from the untranslated regions (UTRs) of well-defined transcriptional units [14]. CRISPR (Clustered regularly interspaced short palindromic repeats) RNA (crRNA) is also a type of small ncRNA that functions in a guide RNA-nuclease system (e.g., CRISPR-Cas9) to maintain bacterial adaptive immunity by protecting the host bacteria from any foreign genetic material, such as those present within phages and plasmids [15]. Commonly found regulatory ncRNAs in bacteria include small RNAs (sRNAs), riboswitches, and RNA thermometers [1,[16], [17], [18], [19]]. However, many examples of protein coding regulatory RNAs have been found to date [[20], [21], [22]]. Here, we provide an overview of gene regulation mechanisms, biological roles, therapeutic importance, and applications of riboswitches, sRNA, and RNA thermometers found in bacteria.

## 2 Regulatory RNAs in bacteria

Regulatory RNAs in bacteria are involved in a vast array of gene regulatory and modulating functions in controlling various biological processes such as nutrient acquisition, responding to stress, virulence, and biofilm formation [18,23]. Scrutinization of intergenic regions (IGRs; sequences bridging gaps in between protein-coding sequences) of bacterial genomes through comparative genomics has revealed numerous such sequence elements [18,24]. These RNAs can either be *cis*-acting or *trans*-acting to regulate cellular processes. Small RNAs (sRNAs) are among the most common of the above regulatory RNAs [18]. Frequently, sRNAs are transcribed independently and bind in *trans* to mRNA targets to regulate the gene expression [13,17,23]. In contrast, some small transcripts act as global regulators by binding to their protein targets to antagonize their function [18,23]. To date, many regulatory sRNAs have been discovered, operating via different mechanisms to repress or activate bacterial gene expression.

In addition to *trans*-acting RNAs, many *cis*-acting regulatory RNAs have also been discovered. These *cis*-acting RNAs are characterized under several groups including riboswitches, RNA thermometers (RNAT) and T-boxes [17,25,26].

### 2.1 small RNAs

In addition to the canonical transcription factors (TFs), sRNAs are found to be major players in global gene regulatory networks. A significant attribute in most regulatory sRNAs is that they are not constitutively expressed, rather respond to environmental variations to modulate gene expression of numerous targets [6,18]. The most extensively studied largest set of sRNA regulators act through base pairing with mRNA targets, resulting in modulation of stability and translation of the target mRNA [12,18]. Here we use specific examples to describe physiological and mechanistic features of different types of sRNA.

Among the first sRNA regulators discovered in bacteria was the RNA I, which is an sRNA of 108 nucleotides in length [27]. This was found to inhibit ColE1 plasmid replication in *E. coli*, by base pairing and stabilizing the RNA that is cleaved to form the primer for replication of the respective plasmid [[27], [28], [29]]. In 1983, another sRNA of 70 nucleotides in size was discovered to be transcribed from the pOUT promoter of the Tn10 transposon [30]. This was found to repress the transposition process by inhibiting translation of the transposase mRNA via base pairing and occluding translational machinery [30]. The 93 nucleotides long *E. coli micF* RNA was the first reported chromosomally encoded sRNA regulator which inhibits translation of its target mRNA encoding OmpF (major outer membrane porin) through a similar mechanism [31]. These earliest sRNA regulators and several others were identified through gel analysis following the radio labelling of total RNA, or through genetic screens favored by their abundance in the bacterial transcriptome studies [32]. However, their prevalence, significance and key roles in gene regulation leading to physiological responses were not initially focused on.

With the rapid discovery of many new bacterial genome sequences, new regulatory sRNAs have been directly detected using techniques such as cloning or microarrays with intergenic probes [33,34]. In bacteria that encode the sRNA helper protein Hfq, pulldowns have enriched for putative regulatory sRNAs [35,36]. Recent approaches such as Cross-linking immunoprecipitation sequencing (CLIP-seq) to discover the target RNAs that interact with global regulator proteins such as CsrA have revealed more novel sRNAs in prokaryotic systems [11]. Transcriptome-wide mapping of sRNA-target pairs through RIL-seq (RNA interaction by ligation and sequencing) has been able to capture novel sRNAs as well as their interactomes [37]. Since base pairing of sRNAs to targets are often mediated by chaperones such as Hfq and ProQ, performing UV crosslinking, ligation, and sequencing of hybrid (CLASH) to uncover Hfq (or ProQ) associated RNA-RNA interactions has also been a popular approach to discover novel sRNA and their targets [38]. The use of differential RNA-seq (dRNA-seq), a method that maps all transcription start sites (TSS) and distinguishes them from processed 5′-ends, has proven powerful to map promoter elements and suggest transcription factor binding sites that allow identification and annotation of novel sRNAs and predictions of conditions under which an sRNAs is active [39]. dRNA-seq alone has contributed to sRNA inventories and transcriptome maps in more than 25 species [40]. Additionally, numerous potential regulatory sRNA sequences in various bacteria have now been predicted through recent advanced techniques such as multilayered computational searches [41,42], deep sequencing, and tiled microarrays with full genome coverage [43]. Up to date, more than 100 sRNA sequences have been identified and verified in *E. coli* through different approaches discussed above (Table 1) [18,44]*.*

**Table 1:** Experimental and predictive approaches used in the discovery of sRNAs and their interactome.

Method	Main findings
1. Co- immunoprecipitation followed by microarray	*E. coli –* Identified 20 novel sRNAs that bind to Hfq including RyaA, and RybC [51]
2. Cross-linking immunoprecipitation sequencing (CLIP-seq)	*E. coli* – Identified 55 sRNAs including AgvB, an abundant Hfq-interacting sRNA [52] *S. Typhimurium*- Identified the binding motifs of sRNA binding proteins Hfq and CsrA [53]
3. UV cross-linking, ligation, and sequencing of hybrids (CLASH)	*E. coli* – Identified mRNA targets of sRNA Esr41 and other interactomes of sRNAs such as tRNA [54]
4. RNA interaction by ligation and sequencing (RIL-seq)	*E. coli* - Identified several novel sRNAs that bind to Hfq Also identified additional targets for established sRNAs [55,56]
5. Differential RNA-seq (dRNA-seq)	*H. pylori* – Identified tmRNA (more than 60 sRNAs studied) [57] *S. typhimurium* – Identified DapZ (around 280 sRNAs studied) [58] *V. cholerae* – Identified VqmR [59]
6. Comparative genome analysis	*P. marinus*- Identified Yfr [60]
7. sRNA identification protocol using high-throughput technologies (SIPHT)	Database search for 932 bacterial replicons yielded 60% confirmed sRNAs [61]
8. Tiling microarray	*L. monocytogenes* – Identified Rli38, RliB and LysRS (50 sRNAs studied) [62]
9. Deep sequencing	*E. coli* – Identified ECS028, ECS031, ECS080, ECS210, ECS026, ECS181, ECS183, ECS224, ECS021, ECS161 (Discovered ∼10 novel sRNAs) [63]

Most of the bacterial regulatory sRNAs characterized to date are synthesized as discrete transcripts with a dedicated promoter and terminator sequences [18,23], or are processed from mRNA 3′ UTRs, though a few 5′ UTR-derived sRNAs have been reported [45]. RNA fragments entirely residing internally in coding sequences also have been suggested to function as regulators [46]. For example, a set of conserved sequences produced by the RNase E activity were identified which are predicted to interact with the RNA chaperones Hfq and ProQ. In addition, using 5′ RNA-seq mapping to search for transcriptional start sites revealed numerous sRNAs transcribed from within coding sequences in several species^,^ [47]. However, the regulatory roles of these sRNAs have not been tested. The characterized sRNAs commonly recognize and bind to 5′ UTR of mRNA and frequently show imperfect complementarity to the target mRNA. As a result of this partial complementarity, a single sRNA can base-pair with multiple mRNA targets and regulate their expression [6]. In many cases, RNA chaperone Hfq aids sRNA-mRNA duplex formation [48]. Hfq remodels RNA to devoid secondary structures and serves as a platform with the increased local concentration of the sRNA and the cognate target mRNA to assess potential complementarity and facilitate interactions [18,23]. With the help of Hfq, annealing to the target can be achieved through only a short and conserved sRNA seed sequence to promote the regulatory activity [49,50]. Some sRNA may even code for proteins [10]. For example, RNAIII, a 514 nucleotides sRNA in *Staphylococcus aureus* base pairs with mRNAs that code for virulence factors and a transcription factor*.* This sRNA also encodes a 26 amino acid delta-hemolysin peptide [18].

#### 2.1.1 Diverse regulatory mechanisms adopted by *trans*-acting sRNA base paring to a target mRNA

**Fig. 1:**
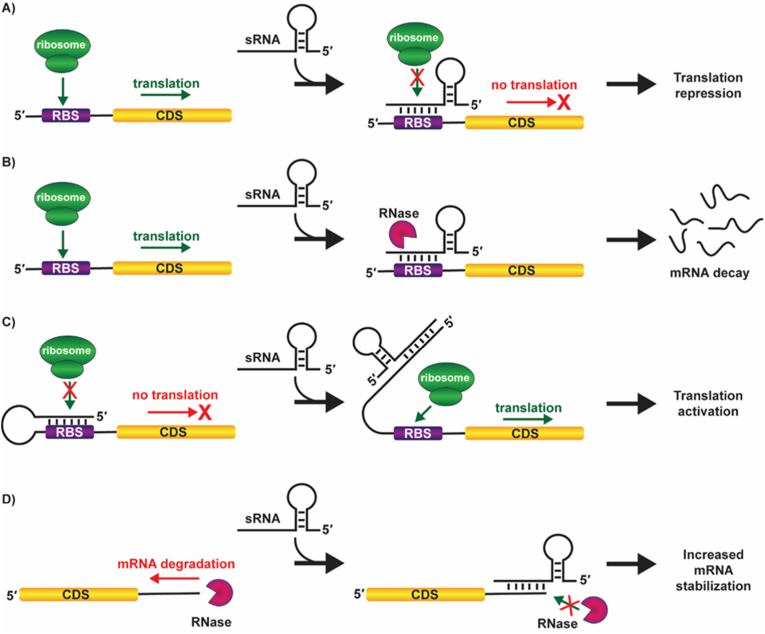
Mechanisms adopted by *trans*-acting sRNA in post-transcriptional gene regulation. **(A)** sRNA binding to 5′ UTR of the target mRNA occludes the ribosome binding site. The ribosomal binding is inhibited, resulting in translation repression. **(B)** sRNA base pairs with an upstream region of the start codon (AUG) of the target mRNA. The sRNA-mRNA duplex is recruited for degradation by an RNase, resulting in robust irreversible repression. **(C)** Positive regulation by an *anti*-antisense mechanism. Binding of sRNA disrupts an inhibitory structure that sequesters the ribosome binding site. Uncovering of the RBS activates translation. **(D)** sRNA binding to the 3′ terminal of the target mRNA prevents the transcript's pre-decay processing and the RNase mediated decay due to the double-stranded form indirectly result in positive regulation by increasing the stable mRNA concentration.

A majority of bacterial sRNA downregulates protein levels via translation inhibition, affecting mRNA stability or through both the mechanisms [1,17]. Frequently, when sRNA binds to the 5′-UTR of the target mRNA, the ribosome binding site is occluded (Fig. 1A). Hence, inhibition of the ribosome binding site and obstruction of the translational machinery become the main contributor to diminished translation levels. For example, in *Salmonella,* GcvB sRNA is targeting several mRNAs and act through the above mechanism. Many of these mRNA targets encode periplasmic substrate-binding proteins of ABC uptake systems for amino acids and peptides [64]. Different mechanisms are adopted by sRNAs such as GcvB and RyhB to downregulate the ABC uptake system's expression. These sRNAs base pair with the far upstream of the transcription start codon (AUG) of the repressed gene [64]. The sRNA-mRNA duplex is eventually degraded by an RNase (*e.g.*, RNase E in *E. coli*) resulting in robust repression and an irreversible negative regulation of the expression of ABC uptake system (Fig. 1B). A similar example of the sRNA binding to the RBS sterically preventing entry of ribosomes and blocking translation is identified in regulation of OmpA synthesis [65]. When present at high levels, the sRNA MicA blocks ribosome binding at the *ompA* translation start site which facilitates the RNase E cleavage and subsequent mRNA decay. MicA requires Hfq protein for this process [65]. Study of the ChiX sRNAs revealed that by base pairing with the 5′ end of its mRNA target in the *chiPQ* operon, the sRNA induces Rho-dependent transcription termination [66]. The mechanism of action involves inhibition of ribosome binding, decreasing ribosome abundance at the Rho utilization site thus facilitating Rho-dependent transcription termination. Cases of transcription termination regulation by sRNAs have been well studied recently [66].

sRNA target sites have also been mapped deep in coding sequence (CDS) regions. *Salmonella* MicC base pairs about 70 nt downstream of the start codon of the ompD mRNA. This interaction has no effect on translation initiation; however, it promotes RNase E-dependent cleavages near the RNA duplex that initiate degradation [67,68].

Positive regulation cases by *trans*-acting sRNA include activation of target mRNA expression through an *anti*-antisense mechanism [69,70]. In some cases, the base pairing of sRNA inhibits Rho dependent termination of the mRNA by preventing Rho from binding to the mRNA [71], while some sRNA act by disrupting an inhibitory secondary structure that sequesters the ribosome binding site [18] (Fig. 1C). DsrA is an 87 nt sRNA in *E. coli*, which is induced at low temperatures. DsrA activates translation by interacting with the protein sigma factor *rpoS* 5′ UTR through an anti-antisense mechanism. This activation results in the upregulation of *rpoS*, which is the central regulator of the general stress response in *E. coli* [72,73]. In addition, GlmZ, RNAIII, RprA, ArcZ, RyhB, and Qrr are other examples of sRNAs that act as direct translational activators by an ‘anti-antisense mechanism’ in the 5′ mRNA region to prevent Rho dependent termination and/or to liberate a sequestered ribosome binding site [70,[74], [75], [76], [77]]. GadY sRNA adopts another mechanism, where its binding to the 3′ UTR alters processing and increases the stability of its target mRNA (Fig. 1D). In some other cases, the same sRNA can act both to repress and activate targets [17].

*Trans*-encoded sRNA is synthesized under specific conditions, for example*,* to regulate genes involved in various biochemical pathways in response to a change in specific environmental conditions. These specific conditions include transition to the stationary phase, nutrient starvation, thermal shock, oxidative stress, and many other environmental challenges. The responsive effects are achieved through modulating metabolic pathways or inducing specific stress responses. Several well-characterized sRNAs and their mechanisms attest the above explanation [6,17]. The RyhB sRNA plays a major functional role in the regulation of iron metabolism in *E. coli* through post-transcriptional regulation of iron metabolism genes [75]. In iron limiting conditions, RyhB promotes rapid degradation of the target mRNAs it pairs with, which is responsible for synthesizing many iron-utilizing proteins, consequently increasing intracellular levels of iron (iron sparing). For example, pairing of RyhB with the target *sodB* induces RNase III-dependent cleavage of RNA-RNA duplex [78]. As a result of iron sparing, the Ferric uptake regulator (Fur) protein is activated, and Fur-regulated genes are repressed. Fur represses the transcription of iron uptake genes by binding to the promoter region of iron-regulated genes with ferric as a cofactor. This basic model, along with a wider network of iron metabolism-related genes controlled by RyhB, integrate into a complex regulatory circuit that maintains iron homeostasis in *E. coli* through direct and indirect negative feedback regulation [75]. In addition to iron homeostasis, other roles for RyhB paralogs were identified in Salmonella [78], such as involvement in nitrate homeostasis [79], oxidative stress, intracellular viability in macrophages, and control of SPI-1 and Type III secretion system gene expression [[80], [81], [82]]. Well-characterized regulatory effects of sRNAs include repression of outer membrane porin proteins under membrane stress conditions (*E. coli* MicA and RybB), and repression of quorum sensing at low density of cells (Vibrio Quorum regulatory RNA) [75,76,83,84]. Additionally, a recent study used a novel MS2 affinity purification coupled with RNA sequencing (MAPS) technique to characterize the targetome of sRNA RsaI in *S. aureus* and identified mRNA targets involved in sugar metabolism, glucose uptake, and biofilm formation, including mRNA transcription factors [85]. RsaI primarily acts as a post-transcriptional repressor on these targets and it is inhibited under high glucose concentrations by CcpA, a global carbon catabolite repressor [85,86].

#### 2.1.2 sRNA binding to proteins

In contrast to the sRNAs that bind to mRNA targets, a group of protein-binding sRNAs (*e.g.*, CsrB, 6S, and GlmY in bacteria) act through a regulatory mechanism that involves antagonizing the activities of their cognate proteins by mimicking the structures of other nucleic acids [9,87,88]. A neat example for this mechanism is the CsrB and CsrC sRNA system that modulates CsrA; an RNA binding protein that regulates carbon utilization, bacterial mobility, and biofilm formation among other roles, upon entry into stationary phase and nutrient-poor conditions [87,89,90]. Once the cells encounter nutrient starvation, transcription of CsrB and CsrC sRNAs is upregulated by a two-component signal transduction system (TCS) BarA-UvrY, which is activated by the products of carbon metabolism such as short chain carboxylate compounds [90,91]. In addition to BarA-UvrY, several other activators regulate CsrB/C transcription in response to nutrient starvation, extra cytoplasmic stress, and other stress conditions. These cellular stressors such as amino acid starvation are detected by the stringent response system, which responds by activating the synthesis of ppGpp, leading to the upregulation of CsrB/C transcription [92,93]. Additional activators of CsrB/C include stringent response factors, DksA, and the two DEAD-box RNA helicases, DeaD (CsdA) and SrmB [89,[92], [93], [94]]. CsrB and CsrC sRNAs mimic the mRNA element (i.e; contains multiple high affinity binding sites for CsrA binding with a GGA motif located in a hexaloop (ARGGAU) of an RNA hairpin) repressed by the CsrA dimer, consequently sequestering CsrA away from mRNA leaders. As a result, CsrA-mediated translation repression and Rho-dependent transcription termination is inhibited, and the mRNA leaders including the ones responsible for carbon usage are available for translation (e.g., *glgC*). These sRNAs simulate an mRNA element and modulates the protein activity [87,89,90,95].

Another set of sRNA found in a wide range of bacteria operates through a mechanism where a DNA element is imitated [9,96]. The 6S RNA in *E. coli* mimics an open promoter, sequester RNA polymerase holoenzyme by binding to transcription factor σ-70 in the enzyme [[96], [97], [98]]. In the stationary phase, 6S RNA transcription is induced, which sequester the σ-70 bound house-keeping form of the RNA polymerase, inhibiting the transcription from certain σ-70 promoters. Since the σ-S bound stationary phase form of RNA polymerase is not sequestered by 6S RNA polymerase, transcription from certain stationary phase-specific σ-S promoters is triggered [97]. The mechanism adopted when the cells exit the stationary phase is that the σ-70/RNA polymerase bound 6S RNA free up to produce a transcript that is responsible for degradation of 6S RNA [88,96,99] consequently making the housekeeping form of the holoenzyme available to transcribe genes with σ-70 promoters. Homologs of this 6S RNA are discovered in a number of organisms [97].

Additionally, GlmY sRNA adopts a mechanism of mimicking another sRNA and works through a protein binding mode of action [9]. GlmY is proposed to act by sequestering or titrating the adaptor protein RapZ away from GlmZ, another sRNA with sequence homology to GlmY [100]. RapZ is an RNA binding protein that recruits RNase E to GlmZ for degradation. Therefore, GlmY expression and its binding to RapZ through a secondary structure shared by both sRNAs frees the homologous GlmZ from RapZ and inhibits the degradation of GlmZ. The full-length GlmZ base pairs with the *glmS* mRNA and activates it for translation, promoting the accumulation of Glucosamine-6-phosphate synthase (GlmS). Thus, GlmY is not only a molecular mimic, but also an anti-adaptor regulating the turnover of another sRNA [100]. These few examples illustrate the approaches of simulating an mRNA element, imitating DNA elements, and mimicking other sRNAs in their regulation mechanisms. They provide an insight into understanding the potential mechanisms of uncharacterized sRNAs and manipulating sRNA for development of RNA-based technologies.

#### 2.1.3 Potential applications of sRNA

The significant physiological roles played by sRNA in regulating key metabolic pathways and stress responses make them attractive for use as tools such as biosensors, negative or positive cell (bacterial) growth controllers, and therapeutic targets [1,23]. Induced sRNA levels in response to a specific condition in the cellular environment could be used as an indicator of the status of the cellular environment. For example, the levels of the RyhB and OxyS sRNAs, respectively, are strong indicators of the iron status and hydrogen peroxide concentration in a cell [8]. Besides, some sRNA involved in stress responses could be targeted and manipulated in such a way to increase the resistance to stress and promote bacterial survival in various industrial and ecological applications [8,17,87].

The regulatory functions of some sRNA are critical for the growth and key metabolic pathways of a vast range of pathogens (*e.g.*, regulation of catabolite repression in *P. aeruginosa* [12], global regulation of quorum-sensing and virulence gene expression in *Staphylococcus aureus* [101] and chromatin condensation in *Chlamydia trachomatis* [102]*.* In many pathogenic bacteria, virulence factors are driven by sRNA regulation [103,104]. For example, sRNA and other regulatory RNAs operate in controlling a single virulence factor, such as the major transcription factor PrfA of the human pathogen *Listeria monocytogenes* [23]*.* Several other examples of sRNAs that positively or negatively control virulence includes RNA III in *Staphylococcus aureus* [103,105], Rli31, Rli33-1, and Rli50 in *L. monocytogenes* [106], IsrM in *Salmonella Typhimurium* [107] and several sRNAs including GlmY, GlmZ and DicF in enterohemorrhagic *E. coli* [108]. Moreover, sRNAs are involved in regulating host-pathogen interactions by promoting bacterial motility and host cell adhesion [109,110]. The ability to deliver sRNAs into host cells from outer membrane vesicles allows modulation of host-pathogen communications and control of the host immune response [111]. Considering the important attributes of sRNA in virulence and pathogenicity, manipulating the respective sRNAs could be an efficacious strategy to combat pathogens. For example, delivery of exogenous antisense oligos targeting these sRNAs can sequester them away from the targets, shutting down a broad spectrum of pathogenic phenotypes. Moreover, sRNA mediated antimicrobial responses and resistance in some bacteria [112,113] can be targeted to address the emergence of bacterial strains resistant to antibiotic treatments. Repression of essential genes and antibiotic resistance genes of pathogens can also be achieved through targeted engineered sRNAs or antisense oligos. On that account, these sRNA regulators could be used as effective targets for antibacterial therapies.

Even in eukaryotes, selected sRNAs could be exploited as novel drug targets to treat and prevent diseases. An interesting approach would be to design sRNAs to base-pair with novel transcript targets of genes associated with the disease or virulence. Since sRNAs can be conveniently modelled, synthesized, and manipulated to attain diverse sequences and structures, rational design and application of sRNA as therapeutic tools are becoming popular approaches [114] Several attributes of sRNA such as the ability to control a wide range of major metabolic transformations, requiring less energy for production compared to proteins (since translation is unnecessary), fast action in post-transcriptional responses, reversibility in action and portability since they can be readily transferred to organisms compared to their protein counterparts, can provide substantial advantages as molecular tools [114].

### 2.2 Riboswitches

Riboswitches are one of the key components of the *cis*-regulatory RNA network found in bacteria. These structured non-coding RNAs are commonly found in 5′ UTRs of mRNAs to regulate gene expression by directly sensing small molecules or ions [16,26,115]. Although transcription and translation are the most common gene-control mechanisms by riboswitches, they have also been shown to regulate gene expression by affecting mRNA splicing, self-cleaving ribozyme activity, and mRNA stability. Unlike common gene-control systems in bacteria such as attenuation systems and protein factor bindings, riboswitches can directly bind to a specific metabolite without a requirement of another RNA or protein factors and control the gene expression [116,117].

**Fig. 2:**
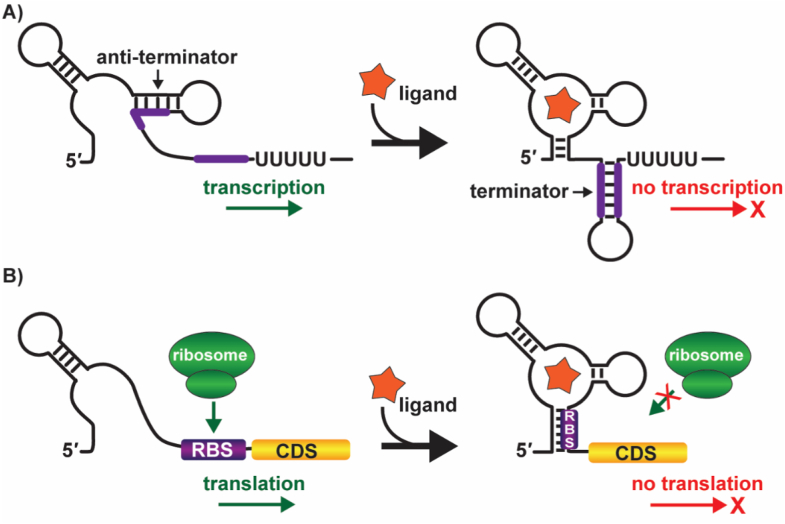
Mechanisms operated by riboswitches to fine-tune gene expression. **(A)** Transcription *anti*-termination and transcription termination: Transcription starts with the formation of the *anti*-terminator loop when the ligand departs and, in the ligand-bound state, the action of RNA polymerase stops when the terminator stem-loop structure is formed. **(B)** Translation activation and inhibition: In the absence of ligand, ribosome binds to the ribosomal binding site (RBS) to start the translational process. Sequestration of the RBS when the ligand is present prevents the translation initiation.

Generally, the riboswitches have two functionally acting modular domains: a highly conserved aptamer region and an expression platform immediately downstream from the aptamer region. The aptamer domain serves as a molecular sensor by selectively binding to the target ligand. Over long periods of evolutionary time, the nucleotide sequence and secondary structure of the aptamer domain remain conserved as the metabolite that is sensed by the riboswitch is unchanged [115,118]. The expression platform transduces the signal to a change in the downstream gene expression by functioning along with the aptamer domain. Upon binding the metabolite to the aptamer region, the expression platform's nucleotides undergo a rearrangement to control gene expression by functioning as ON/OFF switches. Riboswitch-mediated gene expression is mostly regulated by controlling transcription termination [119] or by sequestering/exposing the ribosomal binding site (RBS), also known as the Shine-Dalgarno (SD) sequence, consequently changing the levels of translation (Fig. 2). Besides, some riboswitches control the gene expression by regulating the stability and splicing of mRNA transcripts [118].

#### 2.2.1 Diversity of known riboswitch classes

Different widespread riboswitch classes have been identified in all domains of life that bind to metabolites in the absence of protein factors [26]. Among the metabolite sensing riboswitches, a large number of riboswitch classes sense ligands derived from RNA nucleotides. These ligands include various coenzymes, nucleotide derivatives, and signaling molecules. Among the riboswitch classes that sense nucleotide derivatives, purine sensing riboswitches were shown to bind either adenine or guanine residues [3,118]. The guanine riboswitches which regulate the protein expression involved in the purine salvage pathway responds to varying concentrations of guanine [3]. The adenine riboswitches operate using two different gene regulatory mechanisms (transcription and translation) even though they bind to the same ligand (2-aminopurine (2 A P) or Adenine) [120].

In addition to purine sensing riboswitches, other riboswitch classes sense several coenzymes that are likely derived from purines [121]. To date, six riboswitch classes have been identified that sense the levels of coenzyme *S*-adenosylhomocysteine (SAH) and/or *S*-adenosylmethionine SAM [[122], [123], [124]]. In addition to *SAM* riboswitches, several other riboswitch classes have also been identified as sensors for RNA-derived coenzymes such as Thiamin pyrophosphate (TPP) [125], flavin mononucleotide (FMN), adenosylcobalamin (AdoCbl), and tetrahydrofolate (THF) [119,121,123,126]. Members of THF riboswitch class were termed as “folE motif” RNAs. The *TPP* riboswitches and their variants sense thiamin (vitamin B1) by binding to its natural phosphorylated derivatives (TMP and TPP) [123,127]. The *FMN* riboswitches usually control the gene expression that encode for flavin mononucleotide biosynthesis and transport proteins [122]. FMN and flavin adenine dinucleotide (FAD) are commonly used as redox cofactors for various enzymes. AdoCbl (coenzyme B12) is known to repress the expression of the genes involved in vitamin B_12_ metabolism through direct binding to *AdoCbl* riboswitches in a metabolite-dependent inhibition manner [119]. At high levels of vitamin B_12_, the *btuB* gene that codes for the vitamin B_12_ transporter is repressed at the post-transcriptional level in *E. coli* and *S. typhimuriumi* [128] by *AdoCbl* riboswitches.

Among all the riboswitch classes, there are four known varying classes of ion-responsive riboswitches which respond to different divalent cations. Mg^2+^-I and Mg^2+^- II riboswitches and *NiCo* riboswitch classes (cooperative binding of Ni^2+^ or Co^2+^) fall under the ion-sensing riboswitch category that binds covalently to selective divalent cation [26,129]. Metal ions like Mg^2+^, which is abundant in cells, play a dual role in riboswitch functions as a sensor and/or an effector molecule. The stability of the three-dimensional structure of a riboswitch can be increased with the magnesium ion binding by helping in the co-transcriptional folding [117,130,131]. The interactions between cations like Mg^2+^ and the phosphate backbone is created in the binding pockets in most of the riboswitch classes [132]. *Mg*^*2+*^*-I* riboswitches also referred to *M-Box* (*ykoK* motif), and *Mg*^*2+*^*-II* riboswitches control the metal ion homeostasis upon binding to magnesium ions [132]. *Mg*^*2+*^*-I* riboswitches are more common in gram-positive bacteria and are relatively complex in function compared to *Mg*^*2+*^*-II* riboswitches [132,133]. From the crystallographic studies, Mg^2+^ ions bound Mg^2+^-I riboswitch's structure revealed that the magnesium ions induce a compact tertiary structure for M-box RNAs to regulate the accessibility of a short nucleotide tract involved in the downstream gene expression [117]. Structural studies have revealed that the riboswitch classes that control the expression of genes with small organic metabolites recognize the ligand through a combination of base stacking, electrostatic, and hydrogen bonding interactions. In contrast, *M-box* riboswitches induce a change in the gene expression when in association with an undefined population of metals, instead of single ligand response [117,134]. The *M-box* riboswitch helps control the magnesium homeostasis in *Bacillus subtilis* by lessening the transcription of *mgtE,* which is an Mg^2+^ transporter gene. *Mg*^*2+*^*-II* riboswitches are found in 5′ UTR of *mgtA* gene that codes for the Mg^2+^ transporters. It has been reported that the RNA-dependent helicase Rho facilitates the transcription termination in the *Mg*^*2+*^*-II* riboswitches from *Salmonella enterica* serovar Typhimurium responding to the Mg^2+^ level [2].

*NiCo* riboswitches bind only to four Ni^2+^ or Co^2+^ ions with high specificity, whereas the Mn^2+^ ion has a weak interaction with the *NiCo* riboswitch binding pocket [129]. The heavy metal toxicity levels of those ions within the cell can be monitored with *NiCo* riboswitches by responding to even small concentration increases [129]. The *YybP-ykoY* motif is another orphan riboswitch class whose cognate ligand was unknown for years [18]. Dambach et al.'s work elaborate on manganese ions' possible interactions with *YybP-ykoY* motif to monitor the metal ion toxicity levels in cells [135]. In *Salmonella*, the 5′ UTR of *mntH* gene, which codes for an Mn^2+^ transporter controls the transcription termination when Mn^2+^ is high in cells [136]. The repression of the *mntH* gene by Mn^2+^ sensing riboswitches, the *czcD* gene regulation by *NiCo* riboswitches [129] and maintaining the levels of MgtA, MgtB, and MgtE by Mg^2+^ responding riboswitches [2] show that riboswitches can sense and respond to various transition metals. Fluoride ion-regulating riboswitch is another class with the characteristic feature of the binding pocket for negative point charges [137]. The fluoride ion fits into the Mg^2+^ cage created by the aptamer phosphate groups to regulate fluoride ions' toxic levels [26].

Apart from riboswitches that sense ions and metabolites, some classes of riboswitches respond to amino acids. Three of twenty amino acids (glycine, lysine, and glutamine) are sensed by the widespread riboswitch classes. In contrast, the rest of the 17 types are indirectly recognized by the T box regulatory elements present in the 5′ region of the genes involved in the amino acid synthesis and aminoacyl-tRNA synthetase [138]. These T-box regulatory elements selectively base pairs with tRNAs deficient in the amino acid at the 3′ end to promote the expression [26].

In contrast to many riboswitch classes that bind to metabolites and directly control translation/transcription of the downstream genes, *glmS* riboswitches regulate the downstream gene expression by functioning as both a self-cleaving ribozyme and a metabolite-responsive riboswitch [124]. The 5′ region of the *glmS* gene in *B. subtilis* encodes for glucosamine-6-phosphate (GlcN6P) synthase [124]. However, *glmS* riboswitch was deficient in regulatory elements needed for transcription attenuation or translation initiation. Furthermore, the binding of GlcN6P (a precursor in peptidoglycan synthesis), activates the *glmS* mRNA self-cleavage at the 5′ end *in vitro* [139]. As in other metabolite sensing riboswitches, the *glmS* ribozyme, the first allosteric ribozyme, also regulates the RNA repression through the formation of a three-dimensional architecture upon activation by the metabolic product (GlcN6P) of the *GlmS* enzyme [140,141]. This class of riboswitches is unique as they form a binding pocket for GlcN6P and use a metal ion cofactor in accelerating the cleavage of target RNA [141,142].

#### 2.2.2 Riboswitches for therapeutic applications

Riboswitches have become potential targets in developing antibiotics due to their ability to sense differential small molecules. To achieve a desired therapeutic application, the aptamer region plays a critical role in riboswitch function. Since the aptamer domains form structured receptors for their cognate ligands that allow them to bind with a high affinity and selectivity, it is plausible to design riboswitch-targeting compounds with reduced off-target effects compared to other generic drugs which binds to RNA hairpin structures. *In vitro* aptamer selection is done by systematic evolution of ligands by exponential enrichment (SELEX) [143]. Furthermore, known riboswitches' crystal structures can further help the rational design of such compounds that specifically target riboswitches [144]. With a better understanding of bacterial riboswitch regulation, synthetic riboswitches can be designed by combining *in vitro* designed aptamers for each specific ligand with regulatory domains.

Several attempts have been made to design antibacterial compounds by targeting riboswitches. Purine and TPP sensing riboswitches have been engineered *in vivo* whereas SAM, FMN and lysine sensing riboswitches have been developed by *in vitro* methods for drug designing [[145], [146], [147]]. In all organisms, TPP is a vital cofactor in many enzymatic reactions. Chemically synthesized thiamine analogues with slight structural differences such as oxythiamine and pyrithiamine can be utilized for thiamine deficiency [148]. Phosphorylated pyrithiamine forms pyrithiamine pyrophosphate (PTPP), inhibiting several bacterial and fungal species [148]. The chemical structure of PTPP is very similar to that of TPP except for the central thiazole ring, which is replaced by a pyridinium ring [149]. It has been shown that PTPP can bind with *TPP* riboswitches and repress the reporter gene expression in bacteria [150]. Thus, the *TPP* riboswitch is proposed to be the cellular target for PTPP [149].

Roseoflavin, an antimicrobial compound, was known to inhibit several Gram-positive bacterial species' growth by repressing riboflavin biosynthesis [151]. Since roseoflavin is a chemical analogue of FMN, it binds to *FMN* riboswitches and downregulates the reporter gene expression in *B. subtilis*. Furthermore, mutations within an FMN aptamer have been observed in roseoflavin-resistant bacteria. Therefore, *FMN* riboswitches are predicted to be the major target for roseoflavin antimicrobial action [151].

Some lysine analogues such as l-aminoethylcysteine (AEC) and DL-4-oxalysine were initially reported to inhibit certain Gram-positive bacterial growth. Later, the lysine riboswitch was identified as the major cellular target for these analogues in *B. subtilis*. Besides, several lysine analogues have been found to inhibit *B. subtilis* growth by binding to lysine riboswitches *in vitro*, resulting in riboswitch-mediated repression of lysine biosynthesis [152]. In a similar study, the 3-D structure of the guanine riboswitch aptamer from *B. subtilis* was used to rationally design compounds that repress bacterial growth [153]. These potential antimicrobial compounds bind the aptamer region of guanine riboswitch *in vitro* with affinities comparable to a natural ligand and inhibit bacterial growth by triggering guanine riboswitch action [154]. Furthermore, carba-GlcN6P, an analogue of glucosamine-6-phosphate, has been identified as a potential antimicrobial compound that triggers the self-cleavage activity of *glm*S riboswitch in *Staphylococcus aureus*.

### 2.3 RNA thermometers

In bacteria, gene expression modulated by temperature variations is controlled by specific RNA thermo sensors known as RNA thermometers (RNAT) found in the bacterial mRNA [155]. These are complex and efficient temperature sensing specific RNA secondary structures, located mostly at 5′ UTR, to hinder the site's ribosomal binding (by forming a hairpin loop) at low temperatures to prevent the translation initiation [155]. Like riboswitches, RNA thermo sensors are *cis* encoded RNA elements that rapidly respond to temperature changes and transduce the signal to regulate gene expression via temperature-induced conformational changes [156].

Some human pathogens overcome the temperature shift effect as it enters the host system by developing these strategies of RNAT [157]. They block the translation at low temperatures (<30 °C) when outside the host and become active with the destabilization of the RNA structure leading to a conformational shift with temperature change after entering the host system [158]. Higher temperatures (∼37 °C) favor RNA thermometers' open structure to initiate the translation by recruiting ribosome to the site and thus promoting the translation of bacterial mRNA inside the host [159]. The RNAT temperature-responsive mechanism was discovered in regulating the heat shock response in *E. coli* [160]. Heat shock and cold shock protein-coding genes and virulence coding genes are highly regulated by thermal sensitive RNAT [161].

Among high-temperature RNA sensors, repression of heat shock gene expression (ROSE) and four uridine thermometers are the most common [162]. Almost all the small heat shock genes are controlled by ROSE elements (Eg: *E. coli* inclusion body-binding protein A gene) [163] whose most of 3′ region of the RNA thermometer element pairs with Shine Dalgarno sequence (SD) to sequester the translation Initiation [161]. In the four-uridine class RNAT, the *agsA* gene encoding the heat shock proteins is managed by alternative sigma factor σ32 [164]. The four uridine residues pair with SD sequences, using the mismatch base pairing that could be reversed as a result of temperature rise [164]. *E. coli* mRNA for cold shock protein A (CspA) is the first discovered cold shock RNATs [165]. They form an array of stem-loop structures at the physiological temperature, which sequesters the SD sequence or the start codon. Nonetheless, at very low temperatures, the start codons will have much less interaction with the stem-loop, and the SD sequences become accessible for the ribosome [166]. Also, CspA protein acts as a chaperone to prevent any cold stabilized RNA structure formation, which is not desirable [166]. Recent study indicates the identification of a thermosensor in the 5′UTR *S. aureus* to undergo post-transcriptional regulation in a temperature dependent fashion. This gene, *cidA* is involved in biofilm formation and pathogen survival. *cidA* thermosensor assists translation at lower temperatures which makes them unique since a typical RNAT functions at higher temperatures [167]. RNA based regulators The RNATs represent a refined mechanism for the temperature-dependent gene expression control and the regulation of pathogenicity and starvation. The molecular understanding of RNAT mechanism still needs more study.

## 3 Computational approaches in the discovery of non-coding RNAs

Due to ncRNAs' prominent roles in many biological processes and diseases, researchers have taken great efforts to discover novel ncRNAs in bacteria and other organisms. Initially, numerous experimental strategies were applied to identify novel ncRNAs in the genomes of model organisms. This approach was known as 'Experimental RNomics' and included techniques such as chemical or enzymatic RNA sequencing, genomic tiling microarrays, 'genomic SELEX′, and full-length complementary DNA (cDNA) cloning in the transcriptomes of organism [168]. In addition to these successful experimental methods, many different computational approaches have also been developed to discover ncRNAs of all size and forms. Previously, software tools such as BLAST [169] BLAT [170] and S Search [171] were used in sequence-based homology detection [171]. Since the RNA sequences are less conserved than their secondary structures, programs like INFERNAL [172], and FastR [173] came into use in structural homology-based detection of ncRNAs [171]. However, RNA motif identification programs like ERPIN have now been developed considering both sequence and structural homology information [171,172,174].

GLobal Automatic Small RNA Search go (GLASSgo) is a fully automated computational approach used to identify sRNA homologs. GLASSgo algorithm performs iterative BLASTN search using the input sRNA sequence, identifying sequences with high pairwise identity (>70%) and thereafter employing secondary structural features in a tree-based clustering approach to identify the homologs whose pairwise identity is between 70% and 52% [175]. One of the highly productive strategies for discovering riboswitches has also involved the use of computer algorithms to carry out comparative sequence analysis of the non-coding DNA portions of bacterial genomes [26]. The DNA databases to be searched are reduced in size by examining only the non-coding regions of each genome, as the ncRNAs usually reside outside of protein-coding regions [176].

Yet challenges in distinguishing novel ncRNA from unrelated sequences that show sequence and structural similarities to the common ncRNAs and the increase in the availability of genomic and metagenomic sequence data have necessitated the improvements in the available computational search algorithms [176]. The *de novo* methods utilize both sequence and structural features derived from known ncRNA for the discovery of new ncRNAs. Recently a *de novo* method known as GC-IGR was used to reveal rare structured ncRNAs in bacteria [176]. The intergenic regions (IGRs) are considered to have properties that are consistent with serving as a template to produce ncRNAs [177]. The riboswitches reside in relatively long IGRs, and these IGRs have a higher percentage of G and C nucleotides, which are preferred by RNAs that form secondary structures [26]. These properties of structured ncRNAs are exploited in the GC-IGR computational analysis pipeline [176].

Stav et al. used GC-IGR computational method to analyze genomes, especially of five bacterial species, to uncover rarely structured ncRNAs in them. One of the striking findings was the discovery of a candidate riboswitch class that responds to an intermediate in the biosynthetic pathway for the coenzyme thiamine pyrophosphate (TPP) [176] and it was later experimentally validated [178]. Researchers from the same lab in 2009 sorted the IGRs of *Pelagibacter ubique* based on length and GC content to identify four novel structured ncRNA motifs, including a novel riboswitch class that selectively responds to the coenzyme SAM [179]. And most recently, GC-IGR approach was used to uncover a new class of riboswitch that regulates gene expression in response to NAD + binding in the species *Streptococcus* by the same group of researchers [180].

Stav et al. suggest that identification of the candidate ncRNA permits convenient prediction of structural models and hypotheses regarding biochemical functions. Further Stav et al. state that comparative sequence analysis can then be subsequently used to increase the number of natural representatives of each motif [176]. Hence this computational approach has been proposed to be used in the discovery of other structured ncRNAs which could serve as targets for the development of new classes of antibacterial agents.

However, considering the time needed for manual analysis and the lack of well-defined techniques such as support vector machine (SVM) in the GC-IGR approach to map genomic regions that are enriched with noncoding RNAs, a bioinformatics pipeline called Discovery of Intergenic Motifs PipeLine (DIMPL) that automates the process of total genome analysis has now been introduced by the Breaker research group [181]. This pipeline consists of 2 stages, where in the initial stage a graph is generated using the IGRs that are extracted from the genome considering its length and %GC and including labels for IGRs from known RNA families. Next, the machine learning algorithm SVM classifier is used identify a contiguous region of a genome's %GC versus length plot, before moving to the most computationally intensive steps [181]. In the second stage homology analysis, secondary structure prediction, statistics and finally the visualization of genetic context are performed to identify the candidate ncRNAs. This approach is thought to accelerate the discovery of novel ncRNAs [181].

## 4 Summary

Regulatory RNAs such as sRNAs, riboswitches and RNAT are found to be playing essential roles in regulating a wide range of cellular processes and physiological responses in bacteria by modulating gene expression through different mechanisms of actions. These include and not limited to regulating transcription and translation initiation, mRNA stability and protein stability as well as protein function. The characterized mechanisms of operation in most of these *cis*-acting and *trans*-acting regulatory elements show that they bring about changes in key metabolic pathways and stress responses with respect to extra and intracellular stimuli. Through many elaborated examples with mechanistic details, we have reviewed how their regulatory models' molecular details widen our knowledge on a previously unveiled paradigm of RNA mediated gene expression regulation. Since these regulatory RNAs have influenced in many key metabolic and physiological response pathways and modulate regulation, they show a promising potential to be manipulated as molecular tools and targets for therapeutics. Targeting intrinsic sRNAs or riboswitches of different systems (e.g., sRNA involved in virulence in pathogenic bacteria) cognate with significant molecular pathways to bring about antimicrobial effects is a general interest approach. Diverse mechanisms, abundance, specific targets and modes of action and the regulatory outcomes on a wide array of cellular responses signify the prominence of regulatory RNA systems in living cells as well as their therapeutic aspects. Additionally, the availability of genome sequences, developments in computational methods and automation in the steps involved in the discovery procedures have made it possible to identify novel ncRNAs which could also serve as new classes of therapeutics.

## Declaration of competing interest

The authors declare that they have no known competing financial interests or personal relationships that could have appeared to influence the work reported in this paper.

## Data Availability

No data was used for the research described in the article.
